# Hemopericardium After Cardiopulmonary Resuscitation—Bedside Drainage with a Triple-Lumen Catheter: A Case Report

**DOI:** 10.5811/cpcem.63687

**Published:** 2026-10-13

**Authors:** Michael Gagliardi, Melissa Luu, Rose-Ann Weick, Rebecca White, Mohammad T. Abboud

**Affiliations:** Hackensack University Medical Center, Department of Emergency Trauma, Hackensack, New Jersey

**Keywords:** pericardiocentesis, hemopericardium, cardiac arrest, case report

## Abstract

**Introduction:**

Hemopericardium from chest compression-related cardiac injury is an underrecognized complication of cardiopulmonary resuscitation, with bleeding risk amplified in anticoagulated patients.

**Case Report:**

A 66-year-old man on apixaban and hemodialysis presented in cardiac arrest. Post resuscitation ultrasound revealed pericardial tamponade. With no pericardiocentesis kit available, a seven French triple-lumen central venous catheter was placed under ultrasound guidance via a subxiphoid approach. Approximately one liter of bloody fluid was drained; the patient died within 24 hours.

**Conclusion:**

Hemopericardium is an underrecognized complication of resuscitation in anticoagulated patients. A triple-lumen central venous catheter offers a familiar bedside drainage alternative when a commercial kit is unavailable.

## INTRODUCTION

Cardiac tamponade is a life-threatening condition caused by fluid buildup in the pericardial space, compressing the heart and causing hemodynamic instability. Hemopericardium arising from chest compression-related cardiac injury is an underrecognized complication of cardiopulmonary resuscitation (CPR), with risk amplified in patients receiving anticoagulation and those with comorbidities such as end-stage renal disease that further predispose to bleeding.^1^,^2^ Emergency physicians should maintain a high index of suspicion for this etiology during and after prolonged resuscitation, because timely recognition guides both mechanical decompression and reversal of anticoagulation. Regardless of etiology, prompt pericardiocentesis allows drainage of this fluid, temporarily stabilizing the patient until definitive treatment, such as a pericardial window, can be performed.^3^,^4^ While preparations are made for definitive intervention, an initial aspiration of as little as 50–100 mL can be sufficient to restore hemodynamic stability.^6^

In large or rapidly accumulating effusions, this approach may be insufficient; repeated aspirations may be needed to keep pace with ongoing fluid accumulation. In such cases, an emergency physician may consider placing a drainage catheter for larger volume, more frequent drainage.^5^ Although commercial kits are available, they are often not accessible in the emergency department (ED), especially in resource-limited settings. Many emergency physicians are also more familiar with central venous catheters, such as triple-lumen catheters, than with specialized kits.

## CASE REPORT

A 66-year-old man with hypertension, diabetes, coronary artery disease status post percutaneous intervention, atrial fibrillation on apixaban, and end-stage renal disease on hemodialysis presented to the ED for cardiac arrest. He had two to three days of abdominal pain that worsened suddenly, leading to collapse, which was witnessed by bystanders who initiated CPR. Emergency medical services performed advanced cardiac life support, and the initial rhythm was noted to be asystole. Return of spontaneous circulation (ROSC) was achieved after 30 minutes of chest compressions and medications, including epinephrine, calcium chloride, sodium bicarbonate, and 250 mL of normal saline. He was intubated in the field using an 8.0-mL endotracheal tube, confirmed by bilateral breath sounds and capnography.

Upon arrival at the ED, the patient again became pulseless, and chest compressions were resumed. He received an additional 3 mg of epinephrine, 1 g of calcium chloride, and 1 ampule of sodium bicarbonate and achieved ROSC after eight minutes of chest compressions. Post-ROSC ultrasound showed a large pericardial effusion and signs of cardiac tamponade.

The patient became increasingly hypotensive, and an emergency pericardiocentesis was planned. Because a pericardiocentesis kit was not immediately available, an 18-gauge spinal needle was used to aspirate pericardial fluid using a 60 mL syringe. Given the large volume of effusion and the likelihood that multiple aspirations might be needed, a seven French (7-Fr) triple-lumen central venous catheter kit was prepared to replace the spinal needle using a guidewire. Using a subxiphoid approach under dynamic ultrasound guidance, 50 mL of bloody fluid was first aspirated through the spinal needle, resulting in immediate hemodynamic improvement. Aortic dissection was a consideration because the aspirate was sanguineous, warranting computed tomography (CT) for further evaluation. The guidewire was threaded through the spinal needle without resistance and visualized within the pericardial space on ultrasound.

After dilating the subcutaneous and muscular tissues, the 7-Fr triple-lumen catheter was placed over the wire. Correct positioning was confirmed by injecting agitated saline through the distal port, which produced echogenic material within the pericardial space without intracardiac opacification. An additional syringe of bloody fluid was drained, and the catheter was sutured in place on the chest wall (Image 1). Chest radiograph showed cardiomegaly with a “water-bottle” cardiac silhouette consistent with pericardial effusion, and the catheter projected appropriately over the cardiac silhouette (Image 2).

During his ED course, the patient experienced several episodes of hypotension that were alleviated by aspirating additional fluid from the central venous catheter. While in the CT scanner being evaluated for aortic dissection as a possible cause of his hemorrhagic pericardial effusion, the patient decompensated into pulseless electrical activity arrest. The emergency physician who accompanied him to the CT suite drained an extra 200 mL of fluid during CPR, and ROSC was achieved. The drainage catheter was connected to a Pleur-Evac on low continuous suction once available, and an additional 620 mL of fluid was drained, with ultrasound showing resolution of the pericardial effusion. The CT confirmed the drain was correctly placed in the pericardium and showed no evidence of aortic dissection (Image 3). The patient received andexanet alfa to reverse anticoagulation after weighing the risks and benefits in the setting of large-volume hemopericardium. He was admitted to the medical intensive care unit in stable but critical condition and ultimately died after 24 hours due to complications related to his illness.

*CPC-EM Capsule*

What do we already know about this clinical entity?

*Cardiac tamponade is treated with urgent pericardiocentesis. Hemopericardium can result from anticoagulation, chest trauma, or cardiopulmonary resuscitation.*

What makes this presentation of disease reportable?

*Hemopericardium and tamponade developed in an anticoagulated patient after resuscitation, illustrating a potentially under-recognized complication of cardiopulmonary resuscitation (CPR).*

What is the major learning point?

*Consider hemopericardium in anticoagulated patients with hemodynamic instability after resuscitation. Point-of-care ultrasound guides rapid identification.*

How might this improve emergency medicine practice?

*Recognize CPR-related hemopericardium early with ultrasound; a triple-lumen catheter can serve as a bedside drainage option when a kit is unavailable.*

## DISCUSSION

The pericardium is a double-layered sac surrounding the heart, normally containing 10–50 mL of serous fluid. A pericardial effusion occurs when excess fluid accumulates within this space, most commonly due to infectious, metabolic, autoimmune, malignant, or traumatic processes. Clinical presentation varies widely, from asymptomatic to cardiac tamponade, a life-threatening condition marked by impaired cardiac filling that can lead to hemodynamic instability and death.^1^–^3^ The primary treatment for tamponade physiology is urgent pericardiocentesis, performed via apical, parasternal, or subxiphoid approaches, to improve right ventricular filling.^6^

In large or rapidly accumulating effusions, draining a small volume (50–100 mL) may not be sufficient to maintain perfusion. In such situations, placing a drainage catheter allows for continuous decompression.^7^,^8^ Commercial pericardial drainage kits are not always easily accessible, especially in urgent or resource-limited settings. The use of central venous catheters for pericardial drainage has been described in rural and resource-limited settings^9^–^11^ and is a familiar bedside option for emergency physicians. In our case, a triple-lumen central venous catheter served as a practical bedside alternative when a commercial pericardiocentesis kit was not immediately available.

The etiology of hemopericardium in this case warrants consideration. The patient was anticoagulated with apixaban for atrial fibrillation in the setting of end-stage renal disease on hemodialysis, a combination associated with higher steady-state drug exposure and increased bleeding risk compared to patients with preserved renal function.^12^ Spontaneous hemopericardium is a rare but increasingly recognized complication of direct oral anticoagulant therapy, with an estimated incidence of 0.02–0.069%, and apixaban has been implicated in a growing case literature.^13^,^14^ The prolonged resuscitation course, however, makes CPR-related cardiac injury the most likely primary or amplifying mechanism. Hemopericardium has been documented in approximately 8–9% of forensic autopsy cases following CPR, and longer chest compression duration has been identified as an independent predictor of thoracic injuries overall.^1^, ^2^

Approximately 38 minutes of cumulative chest compressions were delivered between the field arrest and the second ED arrest before tamponade was identified. This case raises the possibility that CPR-induced thoracic trauma, which causes pericardial and myocardial injury in up to 17% of autopsy cases,^1^ may be sufficient to produce clinically significant hemopericardium in fully anticoagulated patients, even in the absence of gross cardiac laceration. Clinicians should maintain a high index of suspicion for pericardial tamponade when return of spontaneous circulation is not achieved, or hemodynamic instability persists following CPR in anticoagulated patients. Whether hemopericardium preceded the initial cardiac arrest or developed as a consequence of prolonged resuscitation in a fully anticoagulated patient cannot be determined with certainty; however, in either scenario, CPR would be expected to worsen pericardial hemorrhage and accelerate progression to tamponade physiology, underscoring the importance of early echocardiographic evaluation during resuscitation of anticoagulated patients.

The use of a central venous catheter as a pericardial drain offers both advantages and limitations in this context. Unlike a pigtail catheter, which has multiple side ports along its curl to maximize drainage, the triple-lumen catheter has a single distal-end hole and two staggered side ports that serve its medial and proximal lumens. These openings are configured for venous infusion rather than fluid drainage, which may increase the risk of obstruction from fibrin, particulate debris, or clotted blood, leading to insufficient flow. Nevertheless, its quick availability and operator familiarity make it a useful temporary option, especially when commercial kits are delayed or inaccessible.

As the population of patients with atrial fibrillation, end-stage renal disease, and chronic direct oral anticoagulant use continues to grow, presentations similar to this case are likely to become more frequent in the ED. Future research should clarify the diagnostic and therapeutic approach to cardiac arrest in anticoagulated patients, including the role of point-of-care echocardiography in early identification of CPR-related hemopericardium, the optimal timing of factor Xa inhibitor reversal during ongoing resuscitation, and the comparative effectiveness of bedside drainage strategies when commercial kits are unavailable.

## CONCLUSION

This case illustrates hemopericardium as an underrecognized complication of CPR in anticoagulated patients. Early identification with point-of-care ultrasound, prompt pericardial decompression, and reversal of anticoagulation are priorities in management; when a commercial pericardiocentesis kit is not immediately available, a triple-lumen central venous catheter provides a familiar bedside drainage alternative.

**TEACHING POINTS**

- Consider hemopericardium in anticoagulated patients with hemodynamic instability after cardiopulmonary resuscitation.
- Use point-of-care ultrasound during and after resuscitation to identify pericardial tamponade as a treatable cause of refractory shock.
- Pursue mechanical decompression and anticoagulation reversal in parallel when tamponade is identified in an anticoagulated patient.
- Use a triple-lumen central venous catheter for bedside pericardial drainage when a commercial pericardiocentesis kit is unavailable.

## Figures and Tables

**Image 1:**
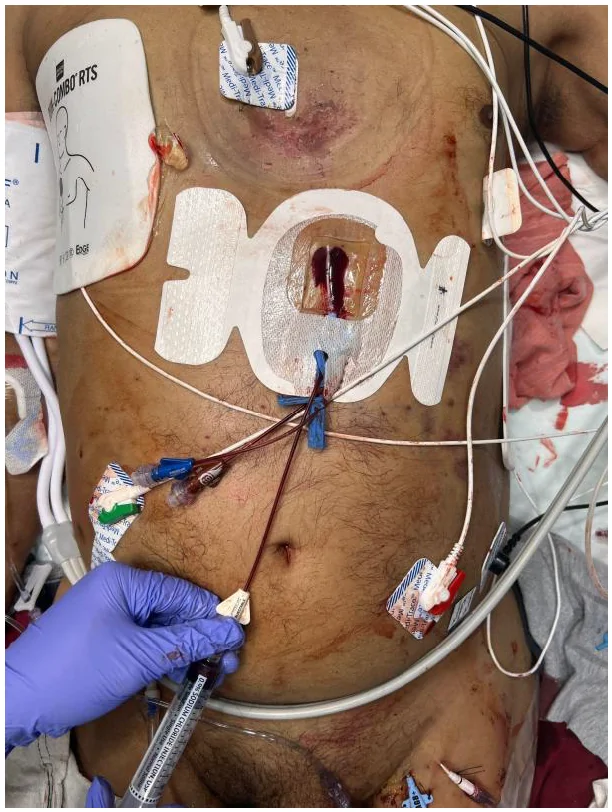
Taken with the family’s permission. A triple-lumen central venous catheter inserted through the chest wall into the pericardium with free-flow aspiration of bloody fluid through a port into a syringe.

**Image 2:**
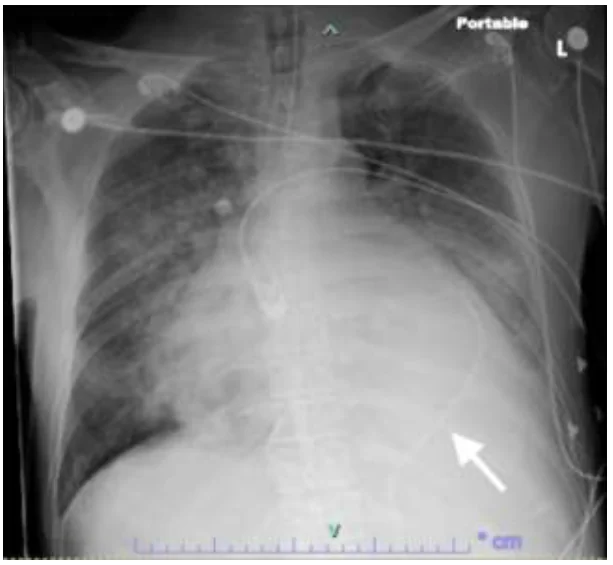
Taken with the family’s permission. A triple-lumen central venous catheter inserted through the chest wall into the pericardium with free-flow aspiration of bloody fluid through a port into a syringe.

**Image 3:**
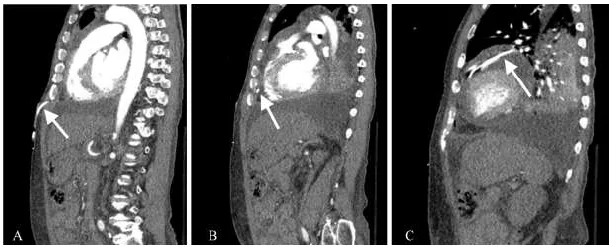
Sagittal computed tomography images confirming the location of the drainage catheter highlighted with white arrows entering the skin via a subxiphoid approach (A), entering the pericardium (B), and within the pericardial space (C).
